# Tetra­kis(μ-2-phenyl­quinoline-4-carboxyl­ato-κ^2^ *O*:*O*′)bis­[(methanol-κ*O*)copper(II)]

**DOI:** 10.1107/S1600536812039839

**Published:** 2012-09-26

**Authors:** Junfang Guo, Guoping Yan

**Affiliations:** a School of Materials Science and Engineering, Wuhan Institute of Technology, 430073 Wuhan People’s Republic of China

## Abstract

The title complex, [Cu_2_(C_16_H_10_NO_2_)_4_(CH_3_OH)_2_], consists of centrosymmetric wheel-shaped dinuclear neutral mol­ecules in which each Cu^II^ atom is coordinated in a slightly distorted square-pyramidal geometry by four O atoms of carboxyl­ate groups from different ligands at the basal plane and an O atom of a methanol mol­ecule at the axial position. In the crystal, the dinuclear complex mol­ecules are linked into one-dimensional supra­molecular columns parallel to the *b* axis by O—H⋯N hydrogen bonds and π–π stacking inter­actions [centroid–centroid distance = 3.7259 (11) Å].

## Related literature

For the background to isonicotinic acid derivatives as polyfunctional ligands, see: Evans & Lin (2002 ▶); Aakeröy *et al.* (1999 ▶); Xiong *et al.* (2000 ▶); Qin *et al.* (2002 ▶); Shen *et al.* (2007 ▶). For the structures of related compounds, see: Bu *et al.* (2005 ▶); Wang *et al.* (2010 ▶); Ma & Lin (2008 ▶).
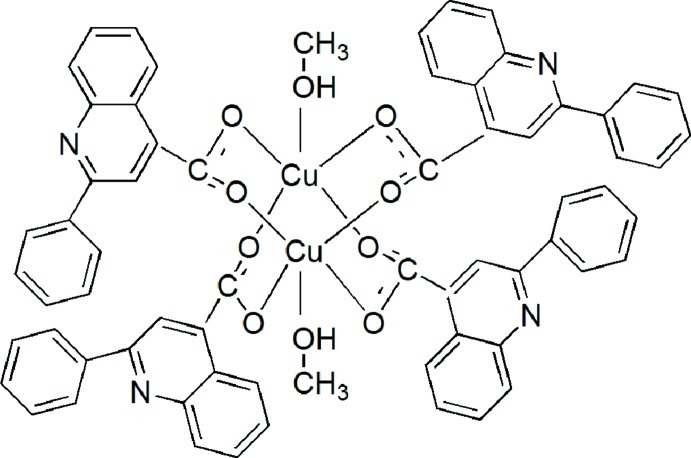


## Experimental

### 

#### Crystal data

[Cu_2_(C_16_H_10_NO_2_)_4_(CH_4_O)_2_]
*M* *_r_* = 1184.16
Triclinic, 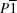

*a* = 8.9671 (6) Å
*b* = 10.5859 (7) Å
*c* = 14.7767 (10) Å
α = 89.800 (1)°
β = 87.348 (1)°
γ = 77.300 (1)°
*V* = 1366.86 (16) Å^3^
*Z* = 1
Mo *K*α radiation
μ = 0.85 mm^−1^
*T* = 293 K
0.31 × 0.24 × 0.17 mm

#### Data collection

Bruker APEX CCD diffractometer
Absorption correction: multi-scan (*SADABS*; Bruker, 2007 ▶) *T*_min_ = 0.780, *T*_max_ = 0.870
7266 measured reflections
4984 independent reflections
4583 reflections with *I* > 2σ(*I*)
*R*_int_ = 0.014

#### Refinement

*R*[*F* ^2^ > 2σ(*F* ^2^)] = 0.033
*wR*(*F* ^2^) = 0.088
*S* = 1.06
4984 reflections
370 parameters
H-atom parameters constrained
Δρ_max_ = 0.48 e Å^−3^
Δρ_min_ = −0.30 e Å^−3^

### 

Data collection: *SMART* (Bruker, 2007 ▶); cell refinement: *SAINT* (Bruker, 2007 ▶); data reduction: *SAINT*; program(s) used to solve structure: *SHELXS97* (Sheldrick, 2008 ▶); program(s) used to refine structure: *SHELXL97* (Sheldrick, 2008 ▶); molecular graphics: *SHELXTL* (Sheldrick, 2008 ▶); software used to prepare material for publication: *SHELXTL*.

## Supplementary Material

Crystal structure: contains datablock(s) I, global. DOI: 10.1107/S1600536812039839/rz5004sup1.cif

Structure factors: contains datablock(s) I. DOI: 10.1107/S1600536812039839/rz5004Isup2.hkl

Additional supplementary materials:  crystallographic information; 3D view; checkCIF report

## Figures and Tables

**Table 1:** Hydrogen-bond geometry (Å, °)

*D*—H⋯*A*	*D*—H	H⋯*A*	*D*⋯*A*	*D*—H⋯*A*
O5—H1⋯N1^i^	0.83	1.95	2.784 (2)	176

Symmetry code: (i) 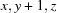.

**Fig. 1.**
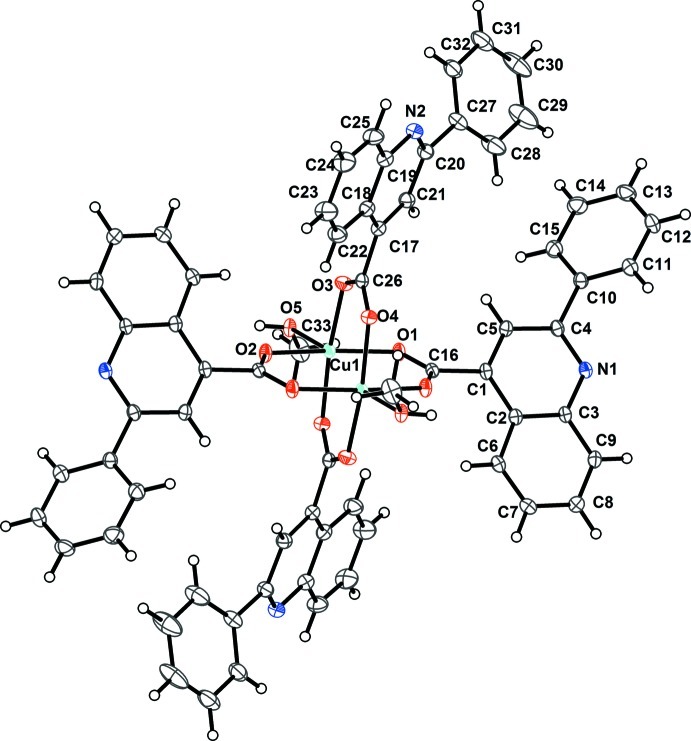
The molecular structure of the title compound, with displacement ellipsoids drawn at the 50% probability level. Unlabelled atoms are related to labelled atoms by the symmetry code (-x+1, -y, -z+1).

**Fig. 2.**
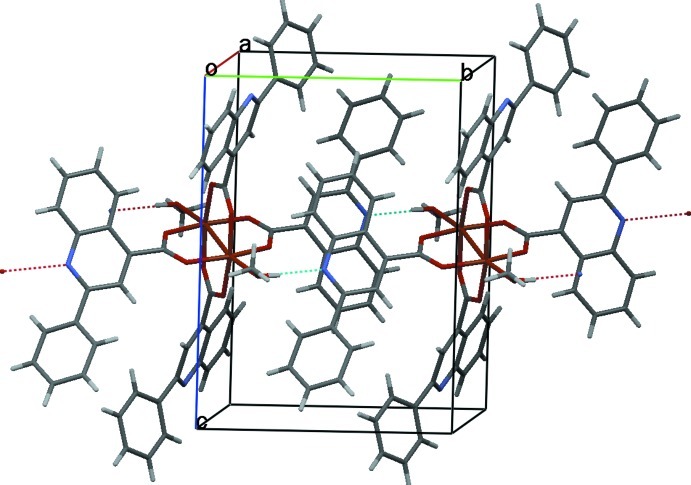
Partial crystal packing of the title compound, showing the formation of a columnar supramolecular structure through hydrogen bonds (dashed lines).

## supplementary crystallographic information

### Comment

Isonicotinic acid and its derivatives, such as 9-acridinecarboxylic acid and 4-quinolinecarboxylic acid, bearing both neutral and anionic donor groups, have been widely used as polyfunctional ligands (Evans & Lin, 2002; Aakeröy *et al.*, 1999; Xiong *et al.*, 2000; Bu *et al.*, 2005). 2-Phenylquinoline-4-carboxylic acid (H*L*), an analogue of isonicotinic acid, exhibits flexible ligation modes in the construction of diverse coordination motifs with unusual properties (Qin *et al.*, 2002; Shen *et al.*, 2007; Wang *et al.*, 2010). Herein, we report a new copper(II) complex derived from 2-phenylquinoline-4-carboxylic acid.

The title complex shows a dinuclear paddle-wheel unit [Cu_2_(*L*)_4_(CH_3_OH)_2_] (Fig. 1), which is composed of two copper(II) ions, four *L* ligands and two methanol molecules. Each metal is pentacoordinated by four O atoms of the carboxylate groups from different ligands [Cu—O mean length = 1.961 (3) Å] at the equatorial plane and one O atom of a CH_3_OH molecule at the axial position. One of the most common parameters used to define the coordination geometry of a pentacoordinated metal center, the τ index, is 0.0003, indicating an almost-ideal square-pyramidal coordination. The metal ion deviates from the mean equatorial plane of the square pyramid toward the apical O5 atom by 0.1942 (3) Å. The Cu···Cu distance is 2.6303 (5) Å, which is within the normal range observed for dinuclear paddle-wheel units in the structures of copper(II) carboxylate complexes (Bu *et al.*, 2005; Wang *et al.*, 2010; Ma & Lin, 2008). In the crystal (Fig. 2), the dinuclear complex molecules are linked into one-dimensional columns parallel to the *b* axis through intermolecular O—H···N hydrogen bonds (Table 1) and π–π stacking interactions involving adjacent quinoline rings, with centroid–centroid distances of 3.7259 (11) Å and perpendicular interplanar separations of 3.4838 (8) Å.

### Experimental

2-Phenylquinoline-4-carboxylic acid (49.8 mg, 0.2 mmol) in CH_3_OH/CHCl_3_ solution (1:1 *v*/*v*, 25 ml) was added to a CH_3_OH solution (25 ml) of Cu(NO_3_)_2_.2.5H_2_O (46.5 mg, 0.2 mmol). The resulting solution was filtered and left to stand at room temperature. Green block-shaped single crystals suitable for X-ray analysis were obtained after several days (yield: 45%).

### Refinement

All H atoms were placed in idealized positions and allowed to ride on their parent atoms, with C—H = 0.93–0.96 Å and O—H = 0.83 Å, and with *U*_iso_(H) = 1.5*U*_eq_(C,O) for methyl and hydroxy H atoms and 1.2*U*_eq_(C) otherwise.

### Crystal data

**Table:**

[Cu_2_(C_16_H_10_NO_2_)_4_(CH_4_O)_2_]	*Z* = 1
*M**_r_* = 1184.16	*F*(000) = 610
Triclinic, *P*1	*D*_x_ = 1.439 Mg m^−^^3^
Hall symbol: -P 1	Mo *K*α radiation, λ = 0.71069 Å
*a* = 8.9671 (6) Å	Cell parameters from 4273 reflections
*b* = 10.5859 (7) Å	θ = 2.7–25.5°
*c* = 14.7767 (10) Å	µ = 0.85 mm^−^^1^
α = 89.800 (1)°	*T* = 293 K
β = 87.348 (1)°	Block, green
γ = 77.300 (1)°	0.31 × 0.24 × 0.17 mm
*V* = 1366.86 (16) Å^3^	

### Data collection

**Table:**

Bruker APEX CCD diffractometer	4984 independent reflections
Radiation source: fine-focus sealed tube	4583 reflections with *I* > 2σ(*I*)
Graphite monochromator	*R*_int_ = 0.014
φ and ω scans	θ_max_ = 25.5°, θ_min_ = 1.4°
Absorption correction: multi-scan (*SADABS*; Bruker, 2007)	*h* = −10→10
*T*_min_ = 0.780, *T*_max_ = 0.870	*k* = −10→12
7266 measured reflections	*l* = −17→14

### Refinement

**Table:**

Refinement on *F*^2^	Primary atom site location: structure-invariant direct methods
Least-squares matrix: full	Secondary atom site location: difference Fourier map
*R*[*F*^2^ > 2σ(*F*^2^)] = 0.033	Hydrogen site location: inferred from neighbouring sites
*wR*(*F*^2^) = 0.088	H-atom parameters constrained
*S* = 1.06	*w* = 1/[σ^2^(*F*_o_^2^) + (0.0453*P*)^2^ + 0.8119*P*] where *P* = (*F*_o_^2^ + 2*F*_c_^2^)/3
4984 reflections	(Δ/σ)_max_ = 0.002
370 parameters	Δρ_max_ = 0.48 e Å^−^^3^
0 restraints	Δρ_min_ = −0.30 e Å^−^^3^

### Special details

**Table:**

Geometry. All e.s.d.'s (except the e.s.d. in the dihedral angle between two l.s. planes) are estimated using the full covariance matrix. The cell e.s.d.'s are taken into account individually in the estimation of e.s.d.'s in distances, angles and torsion angles; correlations between e.s.d.'s in cell parameters are only used when they are defined by crystal symmetry. An approximate (isotropic) treatment of cell e.s.d.'s is used for estimating e.s.d.'s involving l.s. planes.
Refinement. Refinement of *F*^2^ against ALL reflections. The weighted *R*-factor *wR* and goodness of fit *S* are based on *F*^2^, conventional *R*-factors *R* are based on *F*, with *F* set to zero for negative *F*^2^. The threshold expression of *F*^2^ > σ(*F*^2^) is used only for calculating *R*-factors(gt) *etc*. and is not relevant to the choice of reflections for refinement. *R*-factors based on *F*^2^ are statistically about twice as large as those based on *F*, and *R*- factors based on ALL data will be even larger.

### Fractional atomic coordinates and isotropic or equivalent isotropic displacement parameters (Å^2^)

**Table:**

	*x*	*y*	*z*	*U*_iso_*/*U*_eq_	
C1	0.3269 (2)	−0.31756 (19)	0.53165 (14)	0.0228 (4)	
C2	0.3333 (2)	−0.40391 (19)	0.45686 (14)	0.0232 (4)	
C3	0.2708 (2)	−0.51467 (19)	0.47299 (14)	0.0232 (4)	
C4	0.2167 (2)	−0.4635 (2)	0.62569 (14)	0.0245 (4)	
C5	0.2661 (2)	−0.3457 (2)	0.61337 (14)	0.0252 (4)	
H5	0.2569	−0.2876	0.6614	0.030*	
C6	0.3893 (2)	−0.3828 (2)	0.36841 (14)	0.0283 (5)	
H6	0.4318	−0.3113	0.3570	0.034*	
C7	0.3817 (3)	−0.4665 (2)	0.29958 (15)	0.0343 (5)	
H7	0.4211	−0.4526	0.2420	0.041*	
C8	0.3147 (3)	−0.5737 (2)	0.31503 (16)	0.0345 (5)	
H8	0.3066	−0.6281	0.2671	0.041*	
C9	0.2618 (2)	−0.5980 (2)	0.39974 (15)	0.0290 (5)	
H9	0.2195	−0.6699	0.4095	0.035*	
C10	0.1622 (2)	−0.5001 (2)	0.71649 (14)	0.0280 (5)	
C11	0.2097 (3)	−0.6262 (2)	0.74798 (16)	0.0352 (5)	
H11	0.2781	−0.6872	0.7126	0.042*	
C12	0.1557 (3)	−0.6609 (2)	0.83149 (17)	0.0424 (6)	
H12	0.1883	−0.7450	0.8524	0.051*	
C13	0.0531 (3)	−0.5706 (3)	0.88392 (17)	0.0448 (6)	
H13	0.0158	−0.5943	0.9397	0.054*	
C14	0.0061 (3)	−0.4456 (3)	0.85373 (17)	0.0437 (6)	
H14	−0.0631	−0.3853	0.8891	0.052*	
C15	0.0617 (3)	−0.4093 (2)	0.77056 (16)	0.0348 (5)	
H15	0.0317	−0.3242	0.7511	0.042*	
C16	0.3837 (2)	−0.19459 (19)	0.52106 (13)	0.0224 (4)	
C17	0.6511 (3)	−0.0701 (2)	0.75173 (14)	0.0278 (5)	
C18	0.7991 (3)	−0.0616 (2)	0.77910 (15)	0.0302 (5)	
C19	0.8346 (3)	−0.0995 (2)	0.86945 (15)	0.0319 (5)	
C20	0.6033 (3)	−0.1580 (2)	0.89854 (15)	0.0312 (5)	
C21	0.5550 (3)	−0.1164 (2)	0.81085 (15)	0.0299 (5)	
H21	0.4578	−0.1209	0.7938	0.036*	
C22	0.9081 (3)	−0.0138 (3)	0.72450 (17)	0.0404 (6)	
H22	0.8868	0.0109	0.6652	0.049*	
C23	1.0445 (3)	−0.0034 (3)	0.75807 (19)	0.0499 (7)	
H23	1.1148	0.0287	0.7216	0.060*	
C24	1.0790 (3)	−0.0408 (3)	0.84710 (19)	0.0492 (7)	
H24	1.1718	−0.0328	0.8694	0.059*	
C25	0.9774 (3)	−0.0891 (3)	0.90132 (17)	0.0419 (6)	
H25	1.0026	−0.1153	0.9599	0.050*	
C26	0.5958 (2)	−0.0336 (2)	0.65834 (14)	0.0261 (4)	
C27	0.5007 (3)	−0.2130 (2)	0.96154 (16)	0.0369 (5)	
C28	0.4088 (4)	−0.2913 (3)	0.9297 (2)	0.0595 (8)	
H28	0.4114	−0.3103	0.8682	0.071*	
C29	0.3129 (4)	−0.3413 (4)	0.9894 (2)	0.0801 (12)	
H29	0.2533	−0.3954	0.9681	0.096*	
C30	0.3061 (4)	−0.3108 (4)	1.0805 (2)	0.0723 (11)	
H30	0.2397	−0.3424	1.1203	0.087*	
C31	0.3971 (4)	−0.2339 (3)	1.1124 (2)	0.0565 (8)	
H31	0.3928	−0.2142	1.1739	0.068*	
C32	0.4952 (3)	−0.1857 (3)	1.05379 (17)	0.0436 (6)	
H32	0.5577	−0.1347	1.0761	0.052*	
C33	0.0245 (3)	0.1999 (3)	0.5450 (2)	0.0491 (7)	
H33A	−0.0514	0.2706	0.5706	0.074*	
H33B	−0.0003	0.1194	0.5629	0.074*	
H33C	0.0268	0.2059	0.4801	0.074*	
N1	0.21783 (19)	−0.54469 (16)	0.55746 (12)	0.0247 (4)	
N2	0.7381 (2)	−0.14840 (18)	0.92751 (12)	0.0331 (4)	
O1	0.29567 (17)	−0.09218 (14)	0.55023 (10)	0.0291 (3)	
O2	0.51543 (16)	−0.20471 (13)	0.48438 (10)	0.0278 (3)	
O3	0.46126 (18)	0.03347 (15)	0.65482 (10)	0.0324 (4)	
O4	0.68533 (17)	−0.07591 (14)	0.59207 (10)	0.0290 (3)	
O5	0.17001 (16)	0.20531 (13)	0.57646 (10)	0.0267 (3)	
H1	0.1821	0.2812	0.5735	0.040*	
Cu1	0.37041 (3)	0.06756 (2)	0.536332 (16)	0.02076 (9)	

### Atomic displacement parameters (Å^2^)

**Table:**

	*U*^11^	*U*^22^	*U*^33^	*U*^12^	*U*^13^	*U*^23^
C1	0.0185 (10)	0.0188 (10)	0.0312 (11)	−0.0041 (8)	−0.0020 (8)	0.0042 (8)
C2	0.0184 (10)	0.0210 (10)	0.0294 (11)	−0.0027 (8)	−0.0018 (8)	0.0023 (8)
C3	0.0211 (10)	0.0192 (10)	0.0289 (11)	−0.0039 (8)	−0.0009 (8)	0.0037 (8)
C4	0.0215 (10)	0.0228 (10)	0.0294 (11)	−0.0054 (8)	0.0007 (8)	0.0041 (9)
C5	0.0249 (10)	0.0215 (10)	0.0300 (11)	−0.0072 (8)	0.0002 (8)	−0.0004 (8)
C6	0.0291 (11)	0.0263 (11)	0.0306 (11)	−0.0087 (9)	0.0003 (9)	0.0064 (9)
C7	0.0412 (13)	0.0353 (13)	0.0270 (11)	−0.0105 (10)	0.0009 (10)	0.0058 (10)
C8	0.0444 (14)	0.0293 (12)	0.0307 (12)	−0.0092 (10)	−0.0045 (10)	−0.0011 (9)
C9	0.0319 (12)	0.0217 (11)	0.0346 (12)	−0.0084 (9)	−0.0024 (9)	0.0014 (9)
C10	0.0296 (11)	0.0281 (11)	0.0295 (11)	−0.0138 (9)	0.0000 (9)	0.0024 (9)
C11	0.0423 (13)	0.0311 (12)	0.0334 (12)	−0.0110 (10)	0.0007 (10)	0.0048 (10)
C12	0.0583 (16)	0.0334 (13)	0.0397 (14)	−0.0194 (12)	−0.0022 (12)	0.0114 (11)
C13	0.0576 (16)	0.0556 (17)	0.0287 (12)	−0.0297 (14)	0.0042 (11)	0.0051 (11)
C14	0.0470 (15)	0.0501 (16)	0.0350 (13)	−0.0152 (12)	0.0101 (11)	−0.0041 (12)
C15	0.0375 (13)	0.0330 (12)	0.0343 (12)	−0.0090 (10)	0.0020 (10)	0.0009 (10)
C16	0.0232 (10)	0.0210 (10)	0.0238 (10)	−0.0066 (8)	−0.0026 (8)	0.0043 (8)
C17	0.0334 (12)	0.0252 (11)	0.0253 (11)	−0.0071 (9)	−0.0021 (9)	−0.0008 (9)
C18	0.0335 (12)	0.0291 (12)	0.0292 (11)	−0.0094 (9)	−0.0018 (9)	−0.0017 (9)
C19	0.0353 (12)	0.0314 (12)	0.0297 (12)	−0.0085 (10)	−0.0021 (9)	−0.0012 (9)
C20	0.0343 (12)	0.0312 (12)	0.0275 (11)	−0.0062 (10)	−0.0012 (9)	0.0023 (9)
C21	0.0323 (12)	0.0311 (12)	0.0277 (11)	−0.0094 (9)	−0.0027 (9)	0.0010 (9)
C22	0.0442 (14)	0.0494 (15)	0.0327 (13)	−0.0213 (12)	−0.0010 (10)	0.0026 (11)
C23	0.0461 (15)	0.0695 (19)	0.0427 (15)	−0.0320 (14)	0.0009 (12)	0.0010 (13)
C24	0.0397 (14)	0.0660 (19)	0.0484 (16)	−0.0247 (13)	−0.0077 (12)	−0.0021 (14)
C25	0.0427 (14)	0.0529 (16)	0.0327 (13)	−0.0145 (12)	−0.0095 (11)	−0.0011 (11)
C26	0.0336 (12)	0.0224 (10)	0.0256 (11)	−0.0129 (9)	−0.0026 (9)	0.0018 (8)
C27	0.0363 (13)	0.0426 (14)	0.0313 (12)	−0.0074 (11)	−0.0031 (10)	0.0125 (10)
C28	0.0622 (19)	0.086 (2)	0.0424 (16)	−0.0414 (17)	−0.0137 (14)	0.0240 (15)
C29	0.070 (2)	0.128 (3)	0.065 (2)	−0.066 (2)	−0.0248 (17)	0.046 (2)
C30	0.0505 (18)	0.112 (3)	0.061 (2)	−0.0313 (19)	−0.0030 (15)	0.049 (2)
C31	0.0649 (19)	0.0587 (18)	0.0397 (15)	−0.0031 (15)	0.0116 (14)	0.0182 (13)
C32	0.0526 (16)	0.0404 (14)	0.0356 (13)	−0.0065 (12)	0.0034 (11)	0.0096 (11)
C33	0.0239 (12)	0.0522 (16)	0.0721 (19)	−0.0098 (11)	−0.0035 (12)	0.0147 (14)
N1	0.0232 (9)	0.0217 (9)	0.0299 (9)	−0.0066 (7)	0.0005 (7)	0.0035 (7)
N2	0.0389 (11)	0.0341 (11)	0.0266 (10)	−0.0081 (9)	−0.0031 (8)	0.0025 (8)
O1	0.0305 (8)	0.0200 (7)	0.0375 (9)	−0.0089 (6)	0.0078 (6)	0.0006 (6)
O2	0.0236 (7)	0.0204 (7)	0.0407 (9)	−0.0085 (6)	0.0020 (6)	0.0017 (6)
O3	0.0341 (9)	0.0381 (9)	0.0232 (8)	−0.0040 (7)	−0.0006 (6)	0.0037 (7)
O4	0.0327 (8)	0.0311 (8)	0.0232 (7)	−0.0068 (7)	−0.0026 (6)	0.0011 (6)
O5	0.0229 (7)	0.0185 (7)	0.0385 (8)	−0.0044 (6)	−0.0001 (6)	0.0005 (6)
Cu1	0.02186 (14)	0.01805 (14)	0.02282 (14)	−0.00599 (10)	0.00180 (9)	0.00185 (9)

### Geometric parameters (Å, º)

**Table:**

C1—C5	1.361 (3)	C20—N2	1.325 (3)
C1—C2	1.428 (3)	C20—C21	1.423 (3)
C1—C16	1.504 (3)	C20—C27	1.485 (3)
C2—C6	1.413 (3)	C21—H21	0.9300
C2—C3	1.421 (3)	C22—C23	1.367 (4)
C3—N1	1.376 (3)	C22—H22	0.9300
C3—C9	1.415 (3)	C23—C24	1.401 (4)
C4—N1	1.325 (3)	C23—H23	0.9300
C4—C5	1.421 (3)	C24—C25	1.367 (4)
C4—C10	1.486 (3)	C24—H24	0.9300
C5—H5	0.9300	C25—H25	0.9300
C6—C7	1.365 (3)	C26—O4	1.256 (3)
C6—H6	0.9300	C26—O3	1.261 (3)
C7—C8	1.409 (3)	C27—C28	1.388 (4)
C7—H7	0.9300	C27—C32	1.391 (3)
C8—C9	1.362 (3)	C28—C29	1.387 (4)
C8—H8	0.9300	C28—H28	0.9300
C9—H9	0.9300	C29—C30	1.380 (5)
C10—C15	1.390 (3)	C29—H29	0.9300
C10—C11	1.394 (3)	C30—C31	1.372 (5)
C11—C12	1.383 (3)	C30—H30	0.9300
C11—H11	0.9300	C31—C32	1.381 (4)
C12—C13	1.383 (4)	C31—H31	0.9300
C12—H12	0.9300	C32—H32	0.9300
C13—C14	1.377 (4)	C33—O5	1.418 (3)
C13—H13	0.9300	C33—H33A	0.9600
C14—C15	1.390 (3)	C33—H33B	0.9600
C14—H14	0.9300	C33—H33C	0.9600
C15—H15	0.9300	O1—Cu1	1.9581 (14)
C16—O1	1.257 (2)	O2—Cu1^i^	1.9674 (13)
C16—O2	1.260 (2)	O3—Cu1	1.9641 (15)
C17—C21	1.363 (3)	O4—Cu1^i^	1.9812 (14)
C17—C18	1.427 (3)	O5—Cu1	2.1125 (14)
C17—C26	1.508 (3)	O5—H1	0.8340
C18—C22	1.416 (3)	Cu1—O2^i^	1.9674 (14)
C18—C19	1.423 (3)	Cu1—O4^i^	1.9812 (14)
C19—N2	1.370 (3)	Cu1—Cu1^i^	2.6303 (5)
C19—C25	1.411 (3)		
			
C5—C1—C2	119.56 (18)	C23—C22—C18	120.7 (2)
C5—C1—C16	119.45 (18)	C23—C22—H22	119.7
C2—C1—C16	120.98 (17)	C18—C22—H22	119.7
C6—C2—C3	118.79 (19)	C22—C23—C24	120.4 (2)
C6—C2—C1	124.56 (18)	C22—C23—H23	119.8
C3—C2—C1	116.57 (18)	C24—C23—H23	119.8
N1—C3—C9	118.14 (18)	C25—C24—C23	120.5 (2)
N1—C3—C2	122.63 (18)	C25—C24—H24	119.8
C9—C3—C2	119.23 (19)	C23—C24—H24	119.8
N1—C4—C5	121.68 (19)	C24—C25—C19	120.7 (2)
N1—C4—C10	117.63 (18)	C24—C25—H25	119.6
C5—C4—C10	120.69 (19)	C19—C25—H25	119.6
C1—C5—C4	120.35 (19)	O4—C26—O3	126.5 (2)
C1—C5—H5	119.8	O4—C26—C17	117.32 (19)
C4—C5—H5	119.8	O3—C26—C17	116.17 (19)
C7—C6—C2	120.5 (2)	C28—C27—C32	119.1 (2)
C7—C6—H6	119.7	C28—C27—C20	120.9 (2)
C2—C6—H6	119.7	C32—C27—C20	120.0 (2)
C6—C7—C8	120.6 (2)	C29—C28—C27	120.2 (3)
C6—C7—H7	119.7	C29—C28—H28	119.9
C8—C7—H7	119.7	C27—C28—H28	119.9
C9—C8—C7	120.4 (2)	C30—C29—C28	120.0 (3)
C9—C8—H8	119.8	C30—C29—H29	120.0
C7—C8—H8	119.8	C28—C29—H29	120.0
C8—C9—C3	120.4 (2)	C31—C30—C29	120.0 (3)
C8—C9—H9	119.8	C31—C30—H30	120.0
C3—C9—H9	119.8	C29—C30—H30	120.0
C15—C10—C11	119.2 (2)	C30—C31—C32	120.4 (3)
C15—C10—C4	120.3 (2)	C30—C31—H31	119.8
C11—C10—C4	120.4 (2)	C32—C31—H31	119.8
C12—C11—C10	120.3 (2)	C31—C32—C27	120.2 (3)
C12—C11—H11	119.8	C31—C32—H32	119.9
C10—C11—H11	119.8	C27—C32—H32	119.9
C11—C12—C13	120.0 (2)	O5—C33—H33A	109.5
C11—C12—H12	120.0	O5—C33—H33B	109.5
C13—C12—H12	120.0	H33A—C33—H33B	109.5
C14—C13—C12	120.2 (2)	O5—C33—H33C	109.5
C14—C13—H13	119.9	H33A—C33—H33C	109.5
C12—C13—H13	119.9	H33B—C33—H33C	109.5
C13—C14—C15	120.2 (2)	C4—N1—C3	118.90 (17)
C13—C14—H14	119.9	C20—N2—C19	118.18 (19)
C15—C14—H14	119.9	C16—O1—Cu1	116.67 (13)
C14—C15—C10	120.0 (2)	C16—O2—Cu1^i^	128.06 (13)
C14—C15—H15	120.0	C26—O3—Cu1	119.04 (14)
C10—C15—H15	120.0	C26—O4—Cu1^i^	125.28 (14)
O1—C16—O2	126.46 (18)	C33—O5—Cu1	122.05 (15)
O1—C16—C1	116.89 (17)	C33—O5—H1	109.7
O2—C16—C1	116.64 (17)	Cu1—O5—H1	112.6
C21—C17—C18	119.5 (2)	O1—Cu1—O3	88.25 (7)
C21—C17—C26	117.53 (19)	O1—Cu1—O2^i^	168.60 (6)
C18—C17—C26	122.98 (19)	O3—Cu1—O2^i^	89.51 (6)
C22—C18—C19	118.7 (2)	O1—Cu1—O4^i^	89.50 (6)
C22—C18—C17	124.8 (2)	O3—Cu1—O4^i^	168.63 (6)
C19—C18—C17	116.4 (2)	O2^i^—Cu1—O4^i^	90.51 (6)
N2—C19—C25	117.6 (2)	O1—Cu1—O5	100.09 (6)
N2—C19—C18	123.4 (2)	O3—Cu1—O5	99.20 (6)
C25—C19—C18	119.0 (2)	O2^i^—Cu1—O5	91.30 (6)
N2—C20—C21	122.3 (2)	O4^i^—Cu1—O5	92.17 (6)
N2—C20—C27	117.7 (2)	O1—Cu1—Cu1^i^	89.46 (4)
C21—C20—C27	120.0 (2)	O3—Cu1—Cu1^i^	87.46 (5)
C17—C21—C20	120.1 (2)	O2^i^—Cu1—Cu1^i^	79.27 (4)
C17—C21—H21	119.9	O4^i^—Cu1—Cu1^i^	81.37 (5)
C20—C21—H21	119.9	O5—Cu1—Cu1^i^	168.47 (4)
			
C5—C1—C2—C6	178.0 (2)	C23—C24—C25—C19	−1.4 (4)
C16—C1—C2—C6	−0.9 (3)	N2—C19—C25—C24	−179.8 (2)
C5—C1—C2—C3	1.4 (3)	C18—C19—C25—C24	1.3 (4)
C16—C1—C2—C3	−177.41 (17)	C21—C17—C26—O4	−131.5 (2)
C6—C2—C3—N1	178.03 (19)	C18—C17—C26—O4	46.7 (3)
C1—C2—C3—N1	−5.2 (3)	C21—C17—C26—O3	46.2 (3)
C6—C2—C3—C9	−2.1 (3)	C18—C17—C26—O3	−135.6 (2)
C1—C2—C3—C9	174.67 (18)	N2—C20—C27—C28	−143.6 (3)
C2—C1—C5—C4	3.3 (3)	C21—C20—C27—C28	37.0 (4)
C16—C1—C5—C4	−177.83 (18)	N2—C20—C27—C32	36.8 (3)
N1—C4—C5—C1	−4.8 (3)	C21—C20—C27—C32	−142.6 (2)
C10—C4—C5—C1	174.91 (19)	C32—C27—C28—C29	0.0 (5)
C3—C2—C6—C7	0.8 (3)	C20—C27—C28—C29	−179.6 (3)
C1—C2—C6—C7	−175.6 (2)	C27—C28—C29—C30	1.5 (6)
C2—C6—C7—C8	1.4 (3)	C28—C29—C30—C31	−1.8 (6)
C6—C7—C8—C9	−2.5 (4)	C29—C30—C31—C32	0.5 (5)
C7—C8—C9—C3	1.2 (3)	C30—C31—C32—C27	1.0 (4)
N1—C3—C9—C8	−179.0 (2)	C28—C27—C32—C31	−1.2 (4)
C2—C3—C9—C8	1.1 (3)	C20—C27—C32—C31	178.4 (2)
N1—C4—C10—C15	−135.3 (2)	C5—C4—N1—C3	1.1 (3)
C5—C4—C10—C15	45.0 (3)	C10—C4—N1—C3	−178.63 (18)
N1—C4—C10—C11	44.0 (3)	C9—C3—N1—C4	−175.89 (19)
C5—C4—C10—C11	−135.8 (2)	C2—C3—N1—C4	4.0 (3)
C15—C10—C11—C12	1.0 (3)	C21—C20—N2—C19	−1.6 (3)
C4—C10—C11—C12	−178.3 (2)	C27—C20—N2—C19	179.0 (2)
C10—C11—C12—C13	0.5 (4)	C25—C19—N2—C20	179.6 (2)
C11—C12—C13—C14	−0.8 (4)	C18—C19—N2—C20	−1.6 (3)
C12—C13—C14—C15	−0.2 (4)	O2—C16—O1—Cu1	0.5 (3)
C13—C14—C15—C10	1.6 (4)	C1—C16—O1—Cu1	179.94 (13)
C11—C10—C15—C14	−2.0 (3)	O1—C16—O2—Cu1^i^	2.0 (3)
C4—C10—C15—C14	177.2 (2)	C1—C16—O2—Cu1^i^	−177.45 (13)
C5—C1—C16—O1	−48.6 (3)	O4—C26—O3—Cu1	5.0 (3)
C2—C1—C16—O1	130.2 (2)	C17—C26—O3—Cu1	−172.51 (13)
C5—C1—C16—O2	130.9 (2)	O3—C26—O4—Cu1^i^	−8.3 (3)
C2—C1—C16—O2	−50.3 (3)	C17—C26—O4—Cu1^i^	169.13 (13)
C21—C17—C18—C22	−179.1 (2)	C16—O1—Cu1—O3	−89.21 (15)
C26—C17—C18—C22	2.7 (4)	C16—O1—Cu1—O2^i^	−10.4 (4)
C21—C17—C18—C19	−1.8 (3)	C16—O1—Cu1—O4^i^	79.64 (15)
C26—C17—C18—C19	179.97 (19)	C16—O1—Cu1—O5	171.75 (15)
C22—C18—C19—N2	−179.2 (2)	C16—O1—Cu1—Cu1^i^	−1.74 (15)
C17—C18—C19—N2	3.3 (3)	C26—O3—Cu1—O1	89.18 (16)
C22—C18—C19—C25	−0.4 (3)	C26—O3—Cu1—O2^i^	−79.64 (16)
C17—C18—C19—C25	−177.9 (2)	C26—O3—Cu1—O4^i^	10.5 (4)
C18—C17—C21—C20	−1.1 (3)	C26—O3—Cu1—O5	−170.88 (15)
C26—C17—C21—C20	177.21 (19)	C26—O3—Cu1—Cu1^i^	−0.36 (15)
N2—C20—C21—C17	3.0 (3)	C33—O5—Cu1—O1	−45.20 (18)
C27—C20—C21—C17	−177.7 (2)	C33—O5—Cu1—O3	−135.05 (17)
C19—C18—C22—C23	−0.4 (4)	C33—O5—Cu1—O2^i^	135.23 (17)
C17—C18—C22—C23	176.8 (2)	C33—O5—Cu1—O4^i^	44.68 (17)
C18—C22—C23—C24	0.4 (4)	C33—O5—Cu1—Cu1^i^	100.3 (2)
C22—C23—C24—C25	0.5 (5)		

Symmetry code: (i) −*x*+1, −*y*, −*z*+1.

### Hydrogen-bond geometry (Å, º)

**Table:**

*D*—H···*A*	*D*—H	H···*A*	*D*···*A*	*D*—H···*A*
O5—H1···N1^ii^	0.83	1.95	2.784 (2)	176

Symmetry code: (ii) *x*, *y*+1, *z*.
